# Complete mitochondrial genome of Yunnan-Guizhou Plateau endemic fish, *Discogobio macrophysallidos*, in China

**DOI:** 10.1080/23802359.2020.1781580

**Published:** 2020-07-02

**Authors:** Yuping Qiu, Jinjin Jin, Guozhu Chen

**Affiliations:** College of Wetlands/National Plateau Wetlands Research Center, Southwest Forestry University, Kunming, China

**Keywords:** *Discogobio macrophysallidos*, mitochondrial geneome, phylogenetic analysis

## Abstract

The complete mitochondrial genome of *Discogobio macrophysallidos* was first determined and analyzed in this work. Its mitochondrial genome is 16,593 bp in length, consisting of 13 protein-coding genes, 22 transfer RNA (tRNA) genes, two ribosomal RNA (rRNA) genes, and a non-coding control region, and its gene order was consistent with other fishes. Phylogenetic analysis showed that *D. macrophysallidos* were clustered with other three species of *Discogobio*. The complete mitogenome of *D. macrophysallidos* provides new molecular data for the further phylogenetic study of the genus of *Discogobio*.

*Discogobio macrophysallidos* is a small fish that belongs to subfamily Labeoninae (Teleostei: Cyprinidae), with special adaptability to rush water environment, and is a Yunnan-Guizhou plateau endemic species in China. In the past, the research on the genus *Discogobio* mostly focused on the comparison of external morphological features, such as the difference of disk and horseshoe-shaped skin fold, but did not involve the origin and phylogenetic research of this genus (Zhou et al. 2005; Zheng and Zhou 2008). Some studies have found that the interspecific morphology of this genus are similar, resulting in that there are heterologous with same name of some species (Zhao et al. 2017). In this study, we determined the complete mtDNA sequence of *D. macrophysallidos* for the first time, and assessed phylogenetic position with Labeoninae.

The sample was captured from Xingyun Lake (24°21′N, 102°46′E), Yunnan, China. Voucher specimens (No. 578) were deposited at the biological museum of Southwest Forestry University. Paired-end reads were sequenced using Illumina MiSeq platform. A total of 2,362,717 paired-end reads were obtained and assembled *de novo*. The tRNAs were confirmed using tRNAscan-SE (Schattner et al. 2005). The complete mitochondrial genome of *D. macrophysallidos* is 16,593 bp (GeneBank Accession No. MT536775), consisting of 22 transfer RNA (tRNA) genes, two ribosomal RNA (rRNA) genes, 13 protein-coding genes, and a control region and the overall GC content was 41.94%, showing 96.51% identities to the *Discogobio longibarbatus* (GenBank: KY465995) (Zheng and Yang 2017). The order and direction of these genes were identical to other Labeoninae fishes (Xue et al. 2016; Zheng and Yang 2017; Tan et al. 2019; Wang et al. 2019).

We used MEGA7 software (Sudhir et al. 2016) to construct a maximum-likelihood tree (with 1000 bootstrap replicates) based on complete mitogenomes of 16 species from Labeoninae subfamily. The phylogenetic tree showed that four species from *Discogobio* genus were clustered a clade with high bootstrap value supported (Figure 1). The complete mitogenome of the *D. macrophysallidos* in this study provides important data in evolutionary analysis of Labeoninae phylogeny.

**Figure 1.**
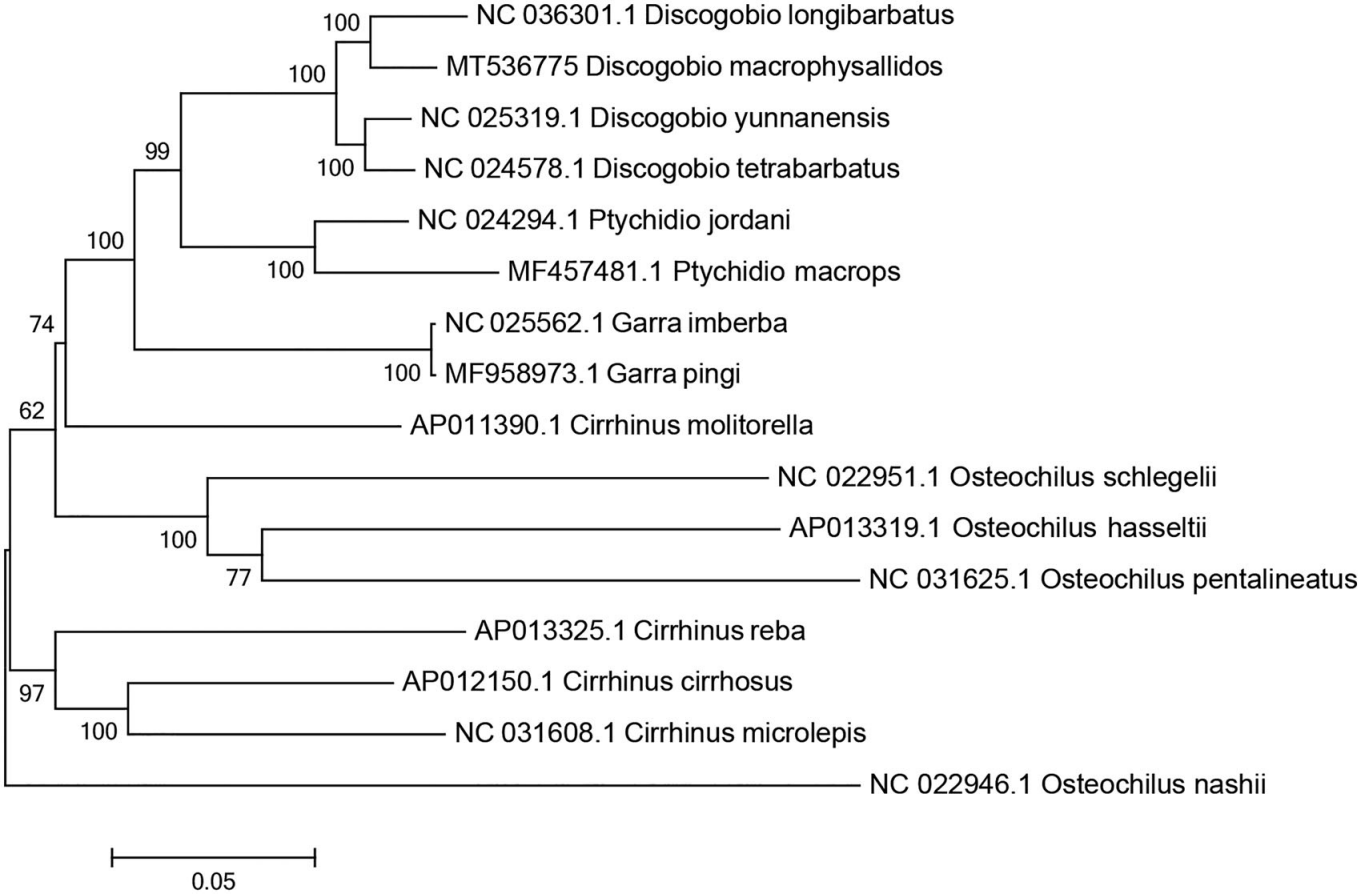
The maximum-likelihood tree of *Discogobio macrophysallidos* and other species based on complete mitogenome using MEGA 7.0.

## Data Availability

The data that support the findings of this study are openly available in NCBI at https://www.ncbi.nlm.nih.gov/nuccore/MT536775, reference numberMT536775.
